# First Report of AIDS-Related Burkitt's Lymphoma Presenting as Bilateral Orbital Masses

**DOI:** 10.1155/2016/6469528

**Published:** 2016-10-13

**Authors:** Monica Alves de Almeida, Juliana Nesi Cardoso Migliano Porto, Ana Carolina de Brito Lyra, Luiz Arthur Calheiros Leite, Rodrigo Panno Basilio-de-Oliveira, Rogério Neves-Motta, Andréa Ramos Correa, Fernando Raphael de Almeida Ferry, Walter de Araujo Eyer-Silva, Ronaldo Grechi Pacheco

**Affiliations:** Hospital Universitário Gaffrée e Guinle, Centro de Ciências Biológicas e da Saúde, Universidade Federal do Estado do Rio de Janeiro, Tijuca, Rio de Janeiro, RJ, Brazil

## Abstract

Burkitt's lymphoma (BL) is an aggressive B-cell non-Hodgkin's lymphoma and one of the fastest growing tumors in humans. It is an acquired immunodeficiency syndrome- (AIDS-) defining disease and occurs with relatively preserved CD4 cell counts. It rarely affects the orbital region in the setting of AIDS. We report unusual presentation of a fatal case of AIDS-associated BL in a 42-year-old female patient with severe CD4 cell depletion who presented with dramatic fast growing (within days) bilateral orbital masses leading to striking facial deformities. To the best of our knowledge, this is the first report of bilateral orbital involvement in AIDS-associated BL.

## 1. Introduction

Non-Hodgkin's lymphomas (NHLs) are the second most common malignancy in patients with the acquired immunodeficiency syndrome (AIDS). Burkitt's lymphoma (BL) is a highly aggressive NHL and is one of the fastest growing tumors in humans [1, 2]. It was first described in 1958 by Burkitt as a mandibular malignancy in African children [3]. BL is classified into three clinical subtypes: (1) endemic BL, which is the most common malignancy of children in equatorial Africa and is associated with low socioeconomic status and antibodies against Epstein-Barr virus antigens; (2) sporadic BL, which affects children and adults in Western countries; (3) immunodeficiency-related BL, which is more common in patients with human immunodeficiency virus (HIV) infection and represents 2.4 to 20% of all AIDS-associated NHLs [1].

Endemic BL is usually multifocal and presents mainly as extranodal jaw or orbital masses. Unlike endemic BL, sporadic BL occurs throughout the world, very rarely involves the jaw or orbits, and commonly presents with abdominal and nodal involvement [1, 2]. Like sporadic BL, AIDS-associated BL typically presents in the gastrointestinal system and/or bone marrow, often with lymph node involvement. Ocular involvement and orbit involvement are very rare [4, 5].

We wish to report unusual presentation of AIDS-associated BL in a 42-year-old female patient with severe CD4 cell count depletion and dramatic fast growing (within days) orbital, eyelid, and frontal masses. To the best of our knowledge, this is the first report of bilateral orbital involvement in AIDS-associated BL.

## 2. Case Report

**Figure 1:**
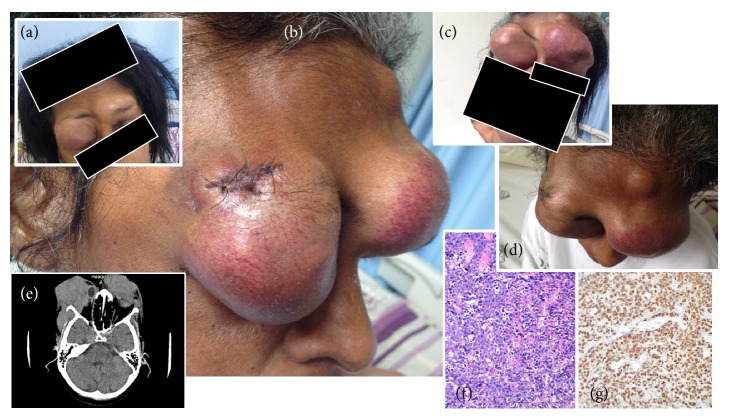
Striking facial deformities due to a dramatic enlargement of orbital and frontal masses between the 3rd (a) and 23rd (b, c, and d) days of hospitalization. CT scan study showing bilateral orbital soft-tissue masses infiltrating and compressing the eyeballs (e). Frontal mass histopathology study (f) presenting diffuse infiltrate of medium-sized malignant lymphoid cells and multiple macrophages with apoptotic debris, creating the “starry sky” pattern (×400; hematoxylin-eosin). Immunochemistry revealed a positive result for Ki-67 > 95% (g).

A 42-year-old HIV-infected female patient, born in and resident of Rio de Janeiro State, Brazil, was admitted to our hospital in August 2015 because of a rapidly growing painful mass in her left shoulder, first noted 4 weeks previously. She also complained of two additional smaller masses over the left eyebrow and right orbits, first noted 3 weeks earlier. The patient was first seen at our outpatient unit in May 2006 due to a diagnosis of HIV infection made in 2003. She had a history of illicit drug use (cocaine). Between 2006 and 2015, she was offered several highly active antiretroviral therapy (HAART) regimens but proved to have poor adherence to treatment. Her current prescribed regimen was lamivudine, tenofovir, atazanavir, and ritonavir. Between 2006 and 2015, the CD4 cell count dropped from 144/mm^3^ to 48/mm^3^ and all 6 plasma viral load measurements yielded very high values (range: 5.4 to 6.7 log), which is suggestive of nonadherence. On examination, the patient appeared to be chronically ill and was oriented and with conjunctival pallor. Vital signs were normal.  Table 1 presents the laboratory data on admission. During the ensuing 20 days of hospitalization, she experienced a dramatic, fast growing enlargement of bilateral orbital, eyelid, and frontal masses (Figure 1). A computed tomography (CT) of the head showed bilateral orbital and frontal masses with osteolytic lesions. The right orbital tumor had infiltrated the optic nerve. An abdominal CT scan revealed hepatomegaly with multiple lesions on the liver parenchyma. Similar lesions were also present on the left kidney, with cortical involvement. Tumors were also evident in the mesenteric lymph nodes. Bone marrow examination was normal. A biopsy of the right orbital mass was consistent with a diagnosis of Burkitt's lymphoma (Figure 1). Immunohistochemistry studies showed positive staining for CD45 (leukocyte common antigen), CD20, and CD10 markers and Ki-67 > 95%, which are expected in Burkitt's lymphoma and reflect the aggressive nature of stage IV B-cell lymphoma. In situ hybridization testing for* c-myc* translocation was not performed. Chemotherapy with EPOCH (etoposide, prednisone, vincristine, cyclophosphamide, and doxorubicin) and filgrastim was started. Her clinical condition, however, worsened. The total white blood cell count dropped to 50/mm^3^ and the patient eventually died of respiratory failure five days later.

## 3. Discussion

AIDS-related lymphomas are comprised almost exclusively of high-grade tumors of B-cells. Among these, BL is the second most common histological subtype. The advent of HAART has not reduced the risk of BL in these patients, despite a decreased risk for other NHLs [1, 6]. BL is composed of monomorphic B-cells with basophilic cytoplasm and numerous mitotic figures that express B-cell antigens such as IgM, CD19, CD20, CD22, and CD79b, germinal center markers such as CD10 and BCL-6, and the proliferation marker Ki-67 in nearly all tumor cells. Moreover, the pathogenesis of BL is due to* c-myc* immunoglobulin (Ig) translocation. The most common translocation is t(8; 14) involving* c-myc* and IgH loci [7]. BL pathophysiology in the context of AIDS is complex and remains unclear. The chronic antigenic stimulation of B-cells induced by HIV leads to deregulation and overexpression of the* c-myc* protein gene, resulting in rapid cell proliferation.

HIV-associated BL most often presents with gastrointestinal and lymph node involvement, in a manner similar to sporadic BL. Orbital, ocular, and eyelid compromises are exceedingly rare. To the best of our knowledge, only four cases of orbital involvement in adult, AIDS-associated BL have been previously described [8–11]. An additional case of BL primarily extending from the right maxillary sinus through the floor of the orbit was reported in a 13-year-old HIV-infected child [12]. All these five cases had unilateral orbital involvement. In contrast, our patient presented with bilateral orbital masses leading to striking facial deformities. We also found evidence of involvement of lymph nodes in the thoracic, abdominal, and pelvic regions, as well as additional frontal and shoulder masses.

It has been shown that the risk of AIDS-associated BL declines steeply at very low CD4 cell counts, suggesting that its expression may require functional CD4 lymphocytes [13]. Therefore, it is surprising that in our patient BL arose in advanced stages of HIV infection, with a CD4 cell count of 48 cells/mm^3^. It is not known whether a different pathogenic trigger exists for the development of BL in patients with advanced HIV infection. No CD4 cell count data is available from previous reports of orbital BL in HIV-infected patients [8–12].

BL can be successfully treated with aggressive chemotherapy. Early recognition and treatment of orbital BL can prevent permanent visual loss and disability. Patients such as ours, with severely depressed CD4 cell count, will pose treatment challenges. The administration of rituximab, for example, was avoided in the present case due to the risk of opportunistic complications.

In summary, physicians who care for HIV-infected patients should be aware that AIDS-associated BL may unusually present as rapidly growing bilateral orbital masses in adult patients with very low CD4 cell counts.

## Figures and Tables

**Table 1:** Laboratory data at admission to the inpatient unit.

Variable	Reference range, adults	At presentation
Hematocrit (%)	36–48	26.1
Hemoglobin (g/dL)	11.5–16.4	8.4
Erythrocyte count (per mm^3^)	4.5–5.9 × 10^6^	2.93 × 10^6^
Mean corpuscular volume (*µ*m^3^)	80–98	89
Mean corpuscular hemoglobin (pg/red cell)	26–34	28.7
Mean corpuscular hemoglobin concentration (pg/red cell)	31–37	32.2
Red-cell distribution width (%)	11.5–14.5	21
Reticulocytes (%)	0.2–2.5	3.8
White-cell count (per mm^3^)	4,000–10,000	7100
Differential count (%)		
Neutrophils	40–70	90
Band forms	0–10	05
Lymphocytes	22–44	03
Monocytes	4–11	02
Basophils	0–1.5	0
Eosinophils	0–8	0
Platelet count (per mm^3^)	15–45 × 10^4^	216
Prothrombin time (sec)	12.2–14.6	10.8
Glucose (mg/dL)	70–100	76
Urea nitrogen (mg/dL)	10–50	20
Creatinine (mg/dL)	0.5–1.2	0.63
Sodium (mmol/L)	135–145	138
Potassium (mmol/L)	3.4–4.8	4.01
Chloride (mmol/L)	100–108	102
Calcium (mg/dL)	8.5–10.5	8.3
Calcium, ionized (mmol/L)	1.14–1.3	1.14
Total protein (g/dL)	6.4–8.3	8.4
Albumin (g/dL)	3.5–5.2	3.7
Globulin	2.5–3.3	4.7
Phosphorus (mg/dL)	2.5–4.5	3.6
Uric acid (mg/dL)	3.5–7	4.74
Alanine aminotransferase (U/L)	0–41	16
Aspartate aminotransferase (U/L)	0–40	26
Alkaline phosphatase (U/L)	30–100	226
*γ*-Glutamyltransferase (U/L)	11–50	179
Total bilirubin (mg/dL)	0.0–1.3	1.20
Direct bilirubin (mg/dL)	0.0–0.3	0.10
Indirect bilirubin (mg/dL)	0.0–0.8	0.10
Amylase (U/L)		173
Lipase (U/L)		137
Lactate dehydrogenase (U/L)	90–250	1421
C-reactive protein (mg/L)	0.0–5.0	208
